# Deform-nu: A DNA Deformation Energy-Based Predictor for Nucleosome Positioning

**DOI:** 10.3389/fcell.2020.596341

**Published:** 2020-12-23

**Authors:** Guoqing Liu, Hongyu Zhao, Hu Meng, Yongqiang Xing, Hui Yang, Hao Lin

**Affiliations:** ^1^School of Life Sciences and Technology, Inner Mongolia University of Science and Technology, Baotou, China; ^2^Center for Informational Biology, University of Electronic Science and Technology of China, Chengdu, China

**Keywords:** deformation energy, rotational positioning, nucleosome occupancy, web server, prediction

## Abstract

The structure and function of chromatin can be regulated through positioning patterns of nucleosomes. DNA-based processes are regulated via nucleosomes. Therefore, it is significant to determine nucleosome positions in DNA-based processes. A deformation energy model was proposed to predict nucleosome positions in our previous study. A free web server based on the model (http://lin-group.cn/server/deform-nu/) was firstly established to estimate the occupancy and rotational positioning of nucleosomes in the study. Then, the performance of the model was verified by several examples. The results indicated that nucleosome positioning relied on the physical properties of DNA, such as deformation energy.

## Introduction

A nucleosome is a histone-DNA complex, in which the histone octamer is wrapped with a ∼147-bp DNA (Kornberg and Lorch, 1999; Richmond and Davey, 2003). A nucleosome regulates DNA-based processes via influencing the proteins’ access to genomic sequences. Thus, it is significant to accurately predict nucleosome positioning. Due to the uncertainty in experimentally determined nucleosome positions caused from cleavage bias in the micrococcal nuclease digestion of chromatin fiber, the precise prediction of nucleosome positioning is extremely important (Flores et al., 2014). Numerous models for predicting nucleosome positions (De Santis et al., 2010; Teif, 2016) are mainly sequence-dependent models. Sequence-based models roughly include two categories of models (De Santis et al., 2010): bioinformatics models and biophysical models. Both have successful application in predicting nucleosome positions. However, the latter is much more interpretable.

We proposed a deformation energy model and successfully predicted occupancy and rotational positioning of nucleosomes with the model (Liu et al., 2016). Based on the model, we also found that bending energy could be used to predict the free energy in nucleosome reconstitution and revealed various patterns of bending energy profile corresponding to different organized chromatin structures, including well-positioned nucleosomes, linker regions, and fuzzy nucleosomes (Liu et al., 2018). In addition, the nucleosome stability was positively correlated with the strength of the bending anisotropy of DNA segment, and directionality and accessibility of nucleosome sliding might be regulated via various patterns of DNA bending energy profile (Liu et al., 2018). In another study, with a machine-learning model, we confirmed that the physical parameters used in the deformation energy model could successfully differentiate nucleosome-enriched regions from nucleosome-depleted regions (Liu et al., 2019). Here, we presented a web server for the deformation energy-based model.

## Deformation Energy Model

We gave a brief introduction to the deformation energy-based model (Liu et al., 2016). Two forms of global deformation of nucleosomal DNA (bending and shear) were considered in the study. We used a 129-bp window in deformation energy calculation for a DNA sequence. In the description of DNA geometry with six degrees of freedom (roll, tilt, twist, shift, slide, and rise) (Dickerson, 1989), elastic energies corresponding to DNA bending and shear are, respectively, formulated as follows:

(1) $$E_{b}\,\left(i\right)=\sum_{i=1}^{128}\left\{\frac{1}{2}\,k_{\rho }\,\left(i\right)\,{\left[\rho \,\left(i\right)-\rho_{0}\,\left(i\right)\right]}^{2}+\frac{1}{2}\,k_{\tau }\,\left(i\right)\,{\left[\tau \,\left(i\right)-\tau_{0}\,\left(i\right)\right]}^{2}\right\}$$

(2) $$E_{s}\,\left(i\right)=\sum_{i=1}^{128}\left\{\frac{1}{2}\,k_{\mathrm{sl}}\,\left(i\right)\,{\left[\mathrm{sl}\,\left(i\right)-{\mathrm{sl}}_{0}\,\left(i\right)\right]}^{2}+\frac{1}{2}\,k_{\mathrm{sh}}\,\left(i\right)\,{\left[\mathrm{sh}\,\left(i\right)-{\mathrm{sh}}_{0}\,\left(i\right)\right]}^{2}\right\}$$

where ρ_*0*_ (i), τ_*0*_ (i), *sh*_*0*_, and *sl*_*0*_ are equilibrium parameters estimated from crystal structures of DNA-protein complexes; ρ(*i*),τ(*i*),*sh*(*i*), and *sl*(*i*) are parameters estimated with two structural constraints (e.g., global curvature and pitch) derived from crystal structures of nucleosomal DNA (Richmond and Davey, 2003); *k*_ρ_ (i), *k*_τ_ (i), *k*_*sh*_ (i), and *k*_*sl*_(*i*) are dinucleotide-dependent force constants estimated with the structures of protein-DNA complexes by inverting the covariance matrix of the six degrees of freedom. The unit of deformation energy is *kT*, where *k* is Boltzmann constant and *T* is effective temperature. After the deformation energy is divided by 128, the number of base-pair steps of the sequence segment, average deformation energy per base-pair step is obtained, whose unit is *kT*/bps, where bps denotes base-pair step.

After the DNA deformation energy is obtained, the probability that a nucleosome dyad is at a site along underlying DNA can be estimated with a grand canonical model (Morozov et al., 2009; Liu et al., 2016), and nucleosome occupancy at a site is the summation of the dyad probabilities of possible nucleosomes covering the site. Nucleosome rotational positioning is predicted with bending energy, whereas the nucleosome occupancy is estimated with shearing energy. Generally, a local minimum of bending energy implicates a high nucleosome dyad probability and the rotational positioning of a nucleosome. In other words, in a nucleosome, the major groove side of DNA at the position with a local deformation energy minimum preferentially faces the histone octamer.

The model differs from previously published models from other groups in the following two aspects: (Richmond and Davey, 2003) global structural constraints (curvature and pitch of the nucleosome super-helix measured from crystal structure) rather than a template nucleosome structure were used in deformation energy calculation; and (Kornberg and Lorch, 1999) because the bending of DNA around histone is largely contributed from roll and tilt and their strong 10-bp oscillation along the nucleosome DNA enables the accurate estimation of bending energy profile with base-pair resolution, only bending energy term is used to predict nucleosome center or rotational positioning.

## Implementation of the Web Server

**FIGURE 1:**
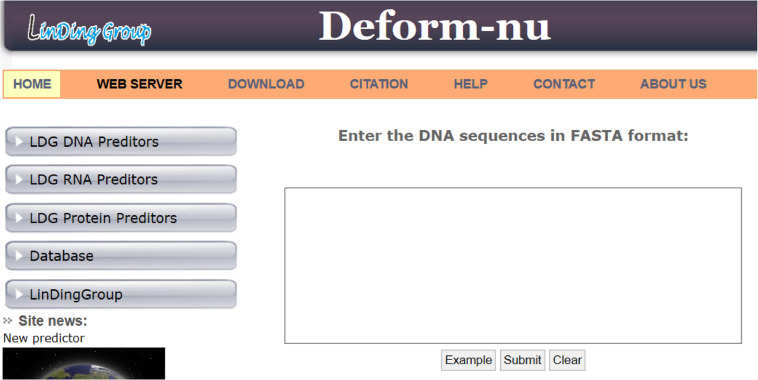
A screenshot of the Deform-nu web server interface.

A free web server (see text foot note 1) was established for our model. Users can obtain DNA deformation energy and nucleosome occupancy after typing or pasting their Fasta formatted sequences in the input box (Figure 1) and clicking the “Submit” button.

Some points of the web server are described below. Firstly, in the reported output for submitted sequences, 147 calculated values of nucleosome occupancy for each end of the sequences are unreliable due to boundary effect. Secondly, in the server, the prediction is carried out by using a pre-defined 129-bp window with a sliding step of 1 bp along the submitted sequence, and all sequences to be predicted should not be shorter than 129 bp. Thirdly, in each submission, it is required that at most 50 sequences are shorter than 50,000 bp. Fourthly, the bending energy and the roll component can be used to predict nucleosome rotational positioning, and the shearing energy is used to calculate nucleosome occupancy. For more details of the web server, see the web server page (see text foot note 1).

## Applications

**FIGURE 2:**
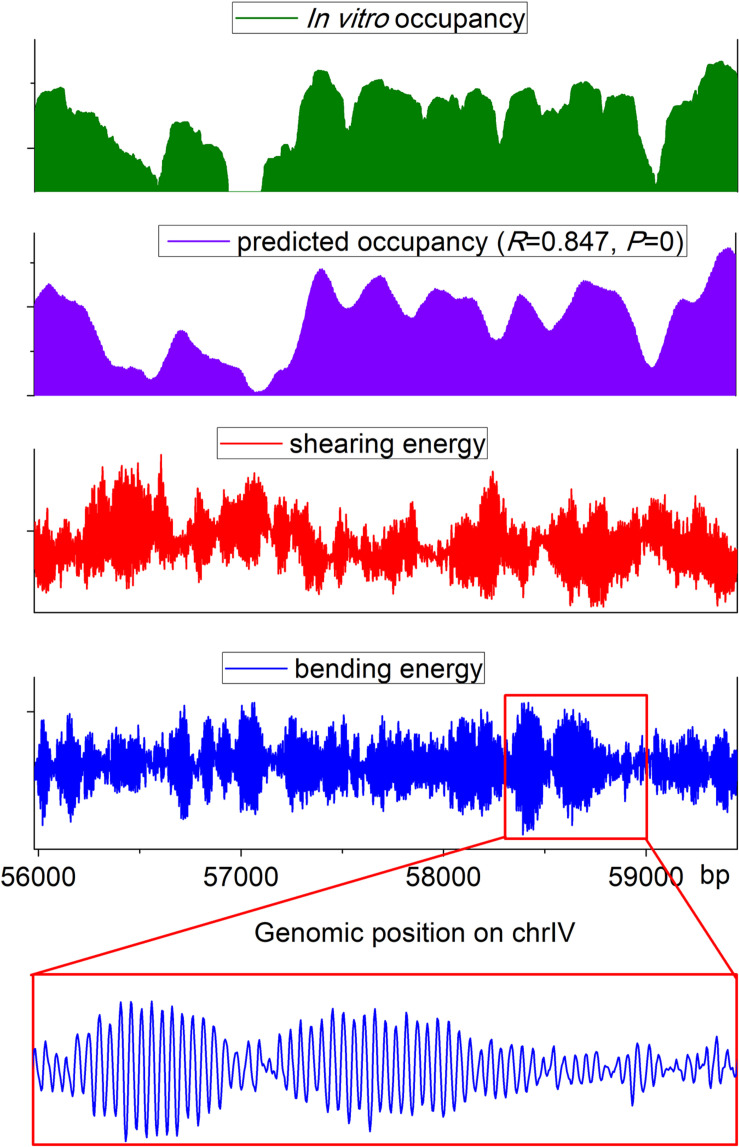
Landscape of estimated nucleosome occupancy, experimental nucleosome occupancy (Kaplan et al., 2009), and corresponding bending energy and shearing energy for a genomic region in budding yeast. The estimated occupancy shows a good agreement with the experimental map. Shearing energy was negatively correlated with the nucleosome occupancy, and the 10-bp oscillated bending energy profile can indicate the rotational positioning of a nucleosome.

The web server presented here has two applications: prediction of nucleosome rotational positioning and nucleosome occupancy. The agreement of the estimated nucleosome occupancy with the *in vitro* nucleosome map (Kaplan et al., 2009) had been demonstrated (Liu et al., 2016) (*R* = ∼0.8, *p* < 0.00001). A nucleosome occupancy landscape estimated for a genomic region is shown along with the experimental nucleosome occupancy, the corresponding bending energy profile, and shearing energy profile in Figure 2. It is evident that our prediction results are highly consistent with an experimental *in vitro* map. The average variation trend of the shearing energy shows a strong negative correlation with the nucleosome occupancy.

**FIGURE 3:**
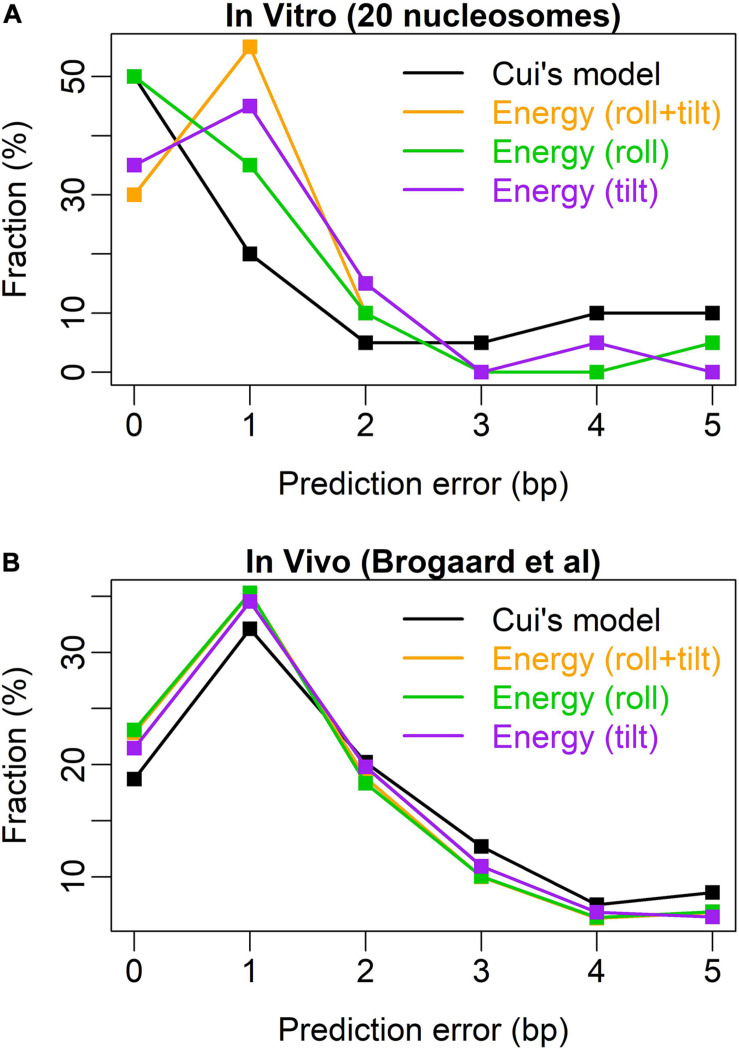
Error distribution for the prediction models. **(A)** Test results of 20 nucleosomes assembled *in vitro* (Cui et al., 2014); **(B)** test results of the *in vivo* map with base-pair resolution (Brogaard et al., 2012). Similar to Cui et al. (2014), the abscissa denotes the prediction error measured by the distance between the experimental nucleosome position and the predicted position with the lowest deformation energy in the interval [-5, +5] around the real nucleosome position. The ordinate denotes the percentage of the error class in the total predicted nucleosome positions.

After testing the model with 20 nucleosomes assembled *in vitro*, we achieved a high prediction performance in nucleosome rotational positioning. Bending energy successfully predicted 19 out of 20 nucleosomes with the uncertainty of no more than 2 bp (Supplementary Figure 1). The prediction performance of the model was better than that of W/S model developed by Cui et al. (2014), the state of the art model. The W/S model failed to predict five nucleosome positions (Cui et al., 2014; Liu et al., 2016), whereas our model successfully predicted four of them (Supplementary Figures 1A–C) and only failed to predict one out-phased nucleosome (position 135 on oocyte 5S rDNA, Supplementary Figure 1I). W/S model precisely predicted (with 0-bp prediction error) more nucleosome positions than bending energy (10 vs. 6). After testing the relative importance of roll and tilt components in bending energy, we found that the roll component precisely predicted 10 out of 20 positions (Figure 3 and Supplementary Figure 1), which equals that of W/S model. Furthermore, the prediction error distribution showed that our prediction model based on bending energy and roll component outperformed W/S model (Figure 3A). Comparing with *in vivo* nucleosome map (Brogaard et al., 2012), we successfully predicted the rotational positioning of ∼77% nucleosomes with the uncertainty of no more than 2 bp, which was better than W/S model (∼70%, Figure 3B). In other words, the rotational positioning of ∼23% nucleosomes in yeasts was not successfully predicted. However, as discussed previously (Liu et al., 2018), it is also possible that at least part of the unsuccessfully predicted nucleosomes might adopt a non-canonical positioning mode *in vivo* in which the major groove side at the dyad position does not face the histones.

**FIGURE 4:**
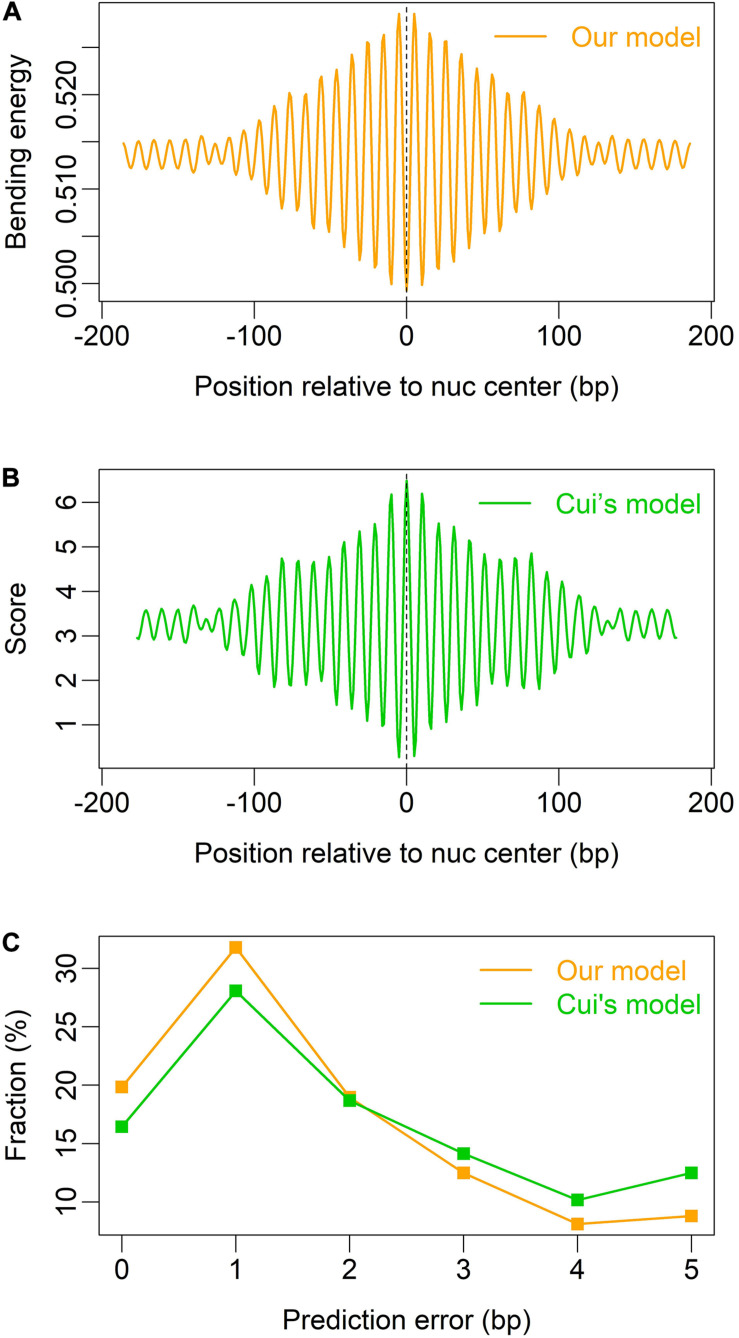
Prediction of nucleosome dyad positions (or rotational positioning) in mouse embryonic stem cells. Non-overlapping nucleosomes with NCP score >2 were analyzed (Voong et al., 2016). **(A)** Local bending energy minima coincide with the experimentally identified nucleosome dyad positions (Voong et al., 2016); **(B)** prediction based on W/S model (Cui et al., 2014); **(C)** comparison of prediction performance between the two models.

We also tested our model on mouse nucleosomes (Voong et al., 2016), and found that both our model and Cui’s model (W/S model) are able to indicate the rotational positioning of nucleosomes in mouse embryonic stem cells (Figures 4A,B), and our model is better than Cui’s model in prediction accuracy (Figure 4C).

**FIGURE 5:**
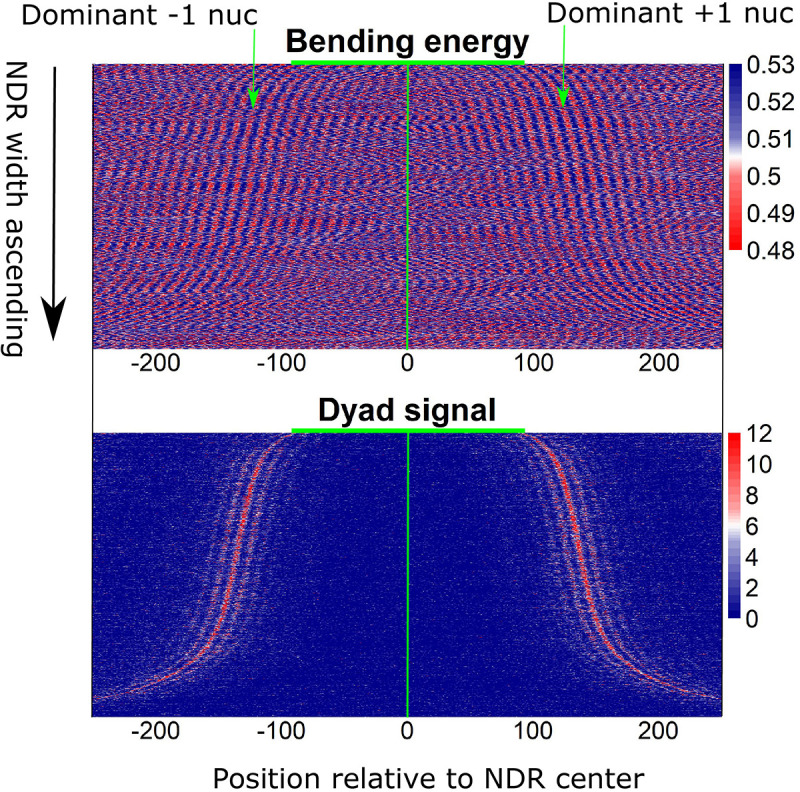
Local bending energy minima coincide with the experimentally identified nucleosome positions around NDRs (Chereji et al., 2018), which differ by multiples of the helical turn and have the same rotational setting.

**FIGURE 6:**
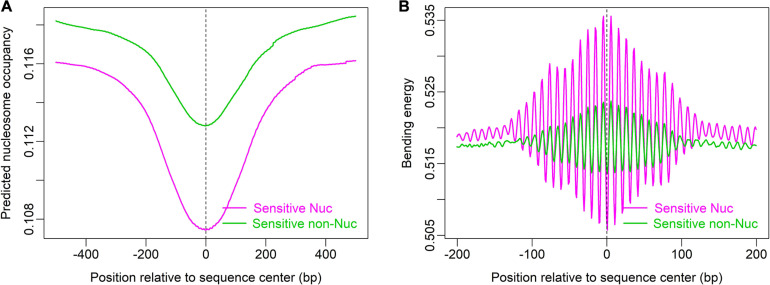
MNase-sensitive nucleosomes have lower predicted nucleosome occupancy than MNase-sensitive non-histone molecules **(A)** but are rotationally more locked than the latter **(B)**. Genomic positions of MNase-sensitive particles were provided by the author of the literature (Chereji et al., 2017).

Furthermore, local bending energy minima coincide with the experimentally identified nucleosome dyad positions around gene upstream nucleosome-depleted regions (NDRs) (Figure 5; Chereji et al., 2018), which differ by multiples of the helical turn and have the same rotational setting. The result further demonstrated the performance of the model in predicting rotational positioning of nucleosomes. The number of experimentally determined dyad signals around dominant +1/-1 nucleosomes was smaller than the number of local bending energy minima observed around the nucleosomes (Figure 5), implicating that although DNA sequence determined the rotational positioning of nucleosome, it was not enough to determine the distribution probability of nucleosomes along the sequence. In addition, we found that although the genomic regions enriched with MNase-sensitive nucleosomes and MNase-sensitive non-histone particles in budding yeast (Chereji et al., 2017) had lower predicted nucleosome occupancy, the regions underlying MNase-sensitive non-histone particles which were located preferentially at promoters were more likely to be occupied by nucleosomes than MNase-sensitive nucleosome regions (Figure 6A). Surprisingly, MNase-sensitive nucleosome regions preferentially located at Transcription Termination Site (TTS) had stronger bending energy oscillation amplitude than MNase-sensitive non-nucleosome molecules (Figure 6B), suggesting that MNase-sensitive nucleosomes had a stronger rotationally locking signal encoded in the DNA sequence. This might be useful in understanding the dynamics and functions of the two kinds of MNase-sensitive complexes.

DNA N6-adenine methylation (6 mA) has recently been described in diverse eukaryotes and plays roles in gene regulation and chromatin organization (Beh et al., 2019). Because of its special site on the DNA base pair, it is conceivable that 6 mA is likely to affect DNA bending and rotational positioning of nucleosomes. Therefore, whether the association between 6 mA and nucleosome rotational positioning can be studied by using our deformation energy model awaits further investigation. In addition, it has been reported that RNAP II pausing signal is stronger at highly phased and highly occupied nucleosomes in mouse embryonic stem cells (Voong et al., 2016), and whether RNAP II pausing also depends on DNA deformation energy profile needs further study. Cancer-related nucleosome alteration (Arimura et al., 2018) is also a possible area where deformation energy modeling may be helpful.

In summary, shearing energy can be used to estimate nucleosome occupancy. The bending energy and its roll component largely guide the rotational positioning of nucleosome: the major groove side of the DNA located at the local energy minima separated with multiples of 10 bp faces the histones. The energy minima also indicated possible dyad positions of the nucleosome. The web server developed here could assist users to infer nucleosome rotational positioning and nucleosome occupancy.

## Data Availability Statement

The original contributions presented in the study are included in the article/Supplementary Material, further inquiries can be directed to the corresponding authors.

## Author Contributions

GL developed the model and carried out the calculation. HZ, HM, and YX performed the data analysis. HL and GL established the web server and wrote the manuscript. All authors contributed to the article and approved the submitted version.

## Conflict of Interest

The authors declare that the research was conducted in the absence of any commercial or financial relationships that could be construed as a potential conflict of interest.
