# Supplementary material for: Deform-nu: A DNA Deformation Energy-Based Predictor for Nucleosome Positioning
Source: Front Cell Dev Biol. 2020 Dec 23;8:596341. doi: 10.3389/fcell.2020.596341 (PMC7785812; doi:10.3389/fcell.2020.596341)
Supplement: Supplementary file 1 [file Data_Sheet_1.PDF]

Supplementary Information for "Deform-nu: a DNA deformation energy-based predictor for nucleosome positioning"

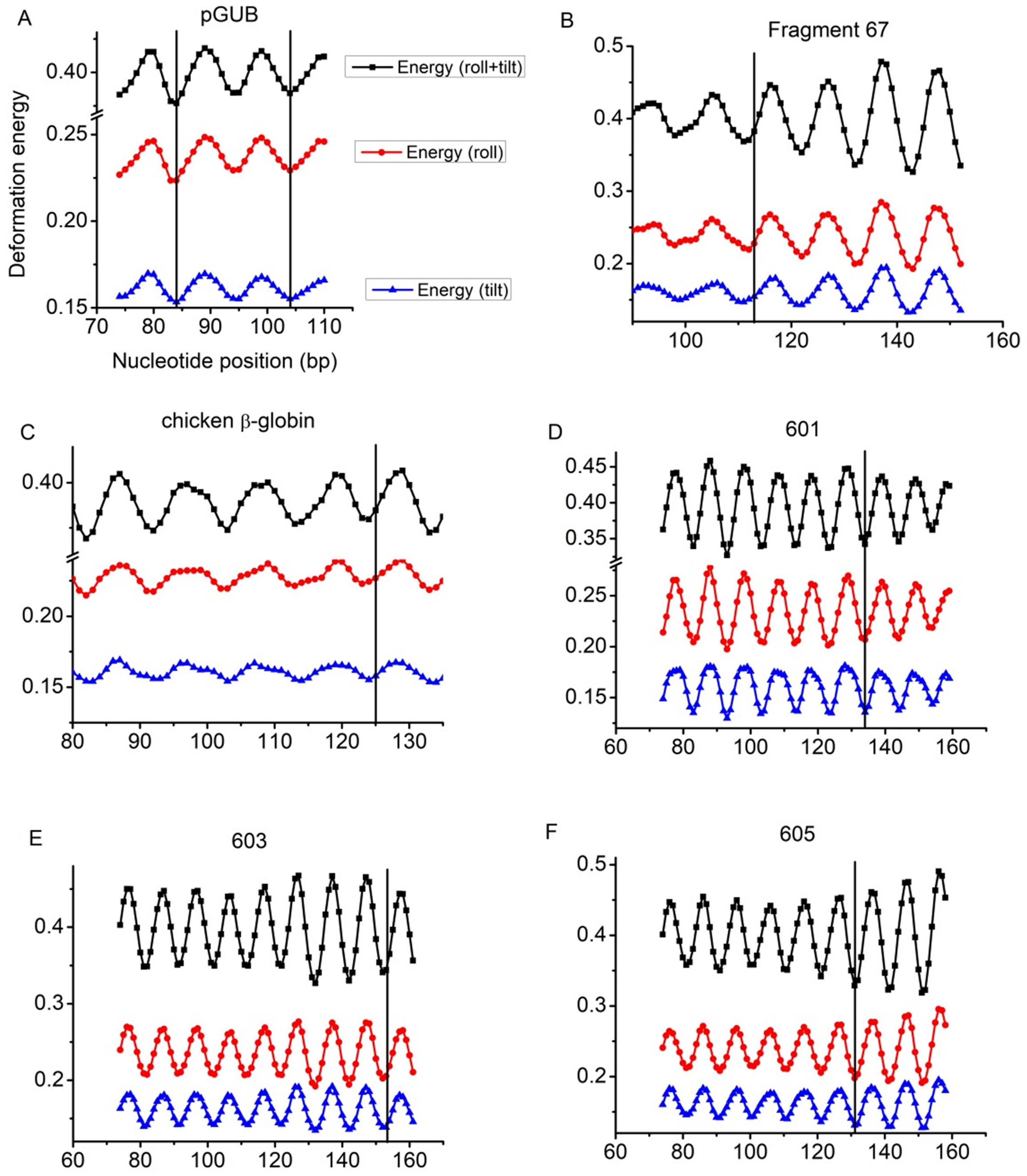

Fig S1 (to be continued)

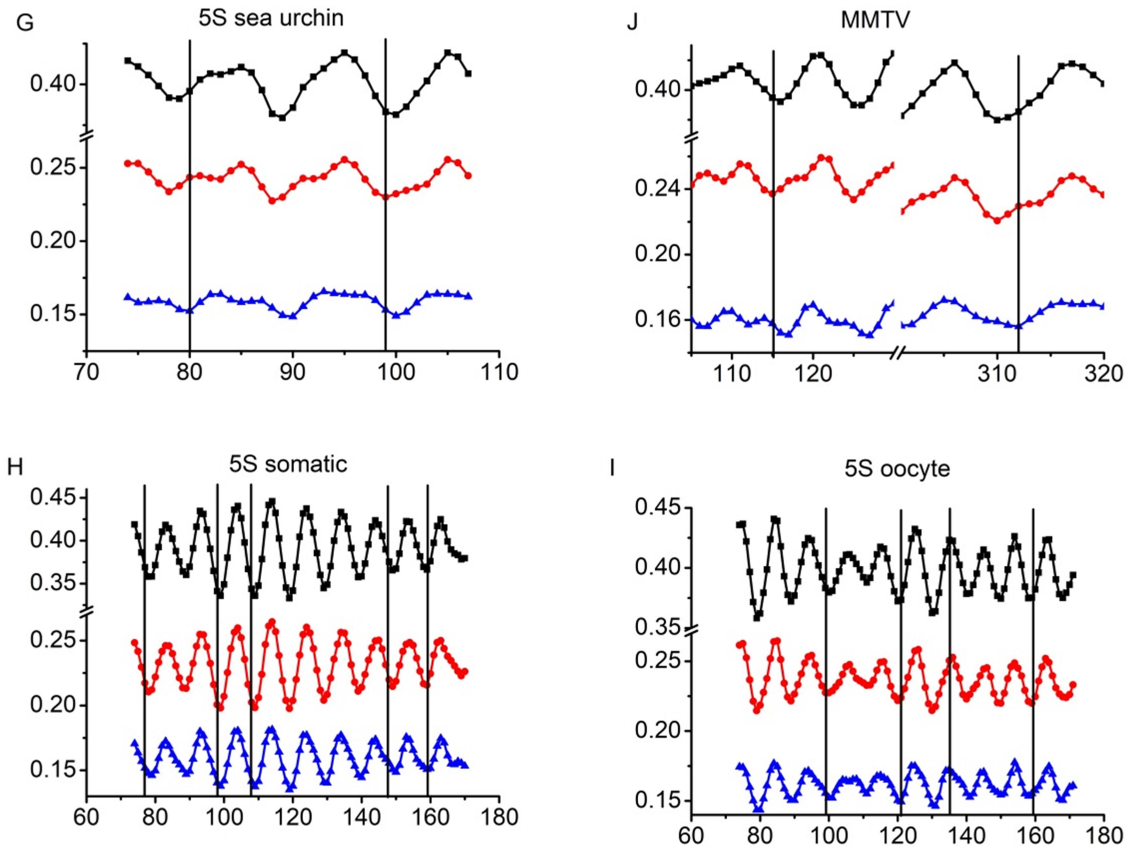

Fig S1. The bending energy (black) and its roll-component (red) and tilt-component (blue) calculated for 10 nucleosomal sequences (20 nucleosomes assembled in vitro on the sequences). The vertical line denotes the experimentally determined dyad positions of the nucleosomes. The W/S model failed to predict 5 nucleosome positions (Cui et al. 2014), while our model only failed for an out-phased nucleosome (position 135 on oocyte 5S rDNA, Fig I). Note that the first nucleotides in the sequences are denoted as nucleotide position 1. The figure legends for all the sub-figures are as in Fig A.
